# Forensic Medicine, Theoretical and Practical

**Published:** 1836-10

**Authors:** 


### Art. V.

*Médecine Légale, Théorique et Pratique, par* Alph. Devergie, Docteur en Médecine, Professeur agrégé de la Faculté de Médecine de Paris, Professeur de Médecine Légale et de Chimie Médicale, &c. *Avec le Texte, et l'Interprétation des Lois relatives à la Médecine, Légale, revus et annotés par* J. B. F. Dehaussy De Robécourt, Conseiller à la Cour de Cassation, &c. Tom. I., Tom. II. P. I.—8vo. pp. 724, 422. *Paris,* 1836.

*Forensic Medicine, Theoretical and Practical, by* Alph. Devergie, &c. *With the Text, and Explanations of the Laws relating to Forensic Medicine, reviewed and commented upon by* J. B. F. Dehaussy Robécourt, &c.

*Elements of Medical Jurisprudence.* By Alfred S. Taylor, f.l.s., Lecturer on Medical Jurisprudence and Chemistry in Guy's Hospital. Vol. I.—*London,* 1836. 8vo. pp. 511.

Forensic medicine is so progressive a science, that the lapse of a very few years is sufficient to render a treatise upon it obsolete, unless new editions are issued in pretty quick succession. The works before us represent the state of the science very fairly up to the present time, but each in its own way, and that way characteristic of the nations to which the authors respectively belong. The French writer goes into his subject with an evident determination to exhaust it; he remains in the mine till he has worked it completely out, apparently caring little whether the fruit of his toil is immediately applicable to any useful purpose. Less profuse of labour, the English author has industry enough to dig up every particle which he thinks may be turned to account, but not an atom more: he may therefore, from inability at the time to calculate their future uses, leave behind him some valuable substances. Mr. Taylor's book, then, is a more practical compendium, as far as it goes; while M. Divergie's far surpasses it in the abundance and variety of its details. Mindful, however, of the long-established character of comparisons, we shall pursue our parallel no farther, but proceed to examine the merits of these works separately.

To any one at all acquainted with the medical literature of France, the name of M. Devergie must be quite familiar. Besides many valuable contributions to the Annales d'Hygiène et de Médecine Légale, he has furnished all the medico-legal articles in the

Dictionnaire de Méd. et Chir. Prat.; a sufficient proof of the consideration in which he is held in his own country. His situation of inspector-physician at the Morgue, (an establishment in which the dead bodies found in the streets and environs of Paris are deposited, in order that they may be identified by their friends,) has afforded him ample opportunities of collecting materials for the elucidation of many intricate questions in forensic medicine. He states, in his preface, that nearly three hundred subjects annually fall under his observation at that institution.

M. Devergie's exordium would lead us to think the definition of Legal Medicine a matter of considerable nicety. He severally criticises the definitions propounded by Mahon and Fodéré, that adopted by Orfila after Prunelle, in the first edition of the Leçons de Méd. Lég., and the altered form which it assumes in the second edition of the latter work; and, having found reason to reject all these, he offers one of his own, which he of course considers exempt from the objections applicable to the others. Mahon and Fodéré define forensic medicine to be the art of applying the principles and maxims of medicine, and its collateral sciences, to the formation of statutes, and to the elucidation and interpretation of certain points of law, (*questions de droit.*) Devergie finds fault with this definition, on the ground that it is not the function of the medical jurist to elucidate or interpret points of law. M. Prunelle's definition runs thus: "Legal medicine is a systematic collection of various physical and medical facts, serving to guide the different orders of the magistracy in the execution and formation of laws." This is open, in our author's judgment, to a twofold charge of incorrectness: 1st. that magistrates are employed in administering justice and enforcing the regulations of police, and that they have nothing to do with the composition of laws; 2dly, that a physician cannot guide the magistrate in the administration of laws, since the application of such and such an article in the law is a *question de droit,* the solution of which, though it must be preceded by certain kinds of information, such as the proof of the *corpus delicti* by the medical practitioner, and the conviction of the criminal by the jury, must, after all, depend upon the judge, who has to determine to what article of the law the crime belongs, and to resolve the different legal questions which arise out of it. We admit the first objection, that magistrates are not *ex-officio* legislators. The force of the second, so far as it bears upon the inability of medicine to assist in the administration of laws, is, we think, without foundation; since, although medicine has nothing to do with the application of the law to a given case, yet, as it establishes the case itself to which the application is to be made, it becomes a necessary, though perhaps a subordinate, instrument in the administration. The more exceptionable part of the definition is, that it specifies magistrates as the officers who are to derive assistance from medical researches; when the persons really aided are those engaged in collecting and proving the facts of a forensic investigation, to which the law is to be applied. Thus, in criminal cases in our own country, the proper function of the judge is to expound the law, and to direct its execution upon a person who has been found guilty by a jury; this decision having been materially influenced by medical testimony. The modification which Orfila's definition underwent in the second edition of his Lectures is as follows: "Legal medicine is a collection of medical facts calculated to throw light upon various points of law, and to direct legislators in the framing of statutes." The latter clause is faultless, but we agree with Devergie that the former is open to the objection already stated. M. Devergie proposes the following as free from all exceptions: "Legal medicine is the art of applying the information derived from physical and medical science to the composition of certain laws, and to the ascertainment and elucidation of certain facts in judicial investigations." In order to illustrate the manner in which medical facts are brought to bear upon juridical enquiries, he traces the progress of a criminal case through the different legal processes; and, as it presents a forcible contrast to the method of investigation pursued in this country, we shall make an abstract of it.

A person having been found dead in his chamber; the Commissaire de police directs a medical practitioner to repair to the spot, and to confirm the fact of the demise and the nature of the death. If there are marks of wounds inflicted during life, the practitioner takes note of them, but without forming any positive conclusion respecting the cause of death: he confines himself to a description of the external appearances, and points out the necessity of opening the body. His report is sent to the Procureur du roi, and, if there are reasons for suspecting unfair play, this officer, or a Juge d'instruction, directs two practitioners to perform the necroscopy, either in his own presence or in that of his deputy. A second report is drawn up, which consists of a statement and explanation of the appearances, and of the conclusions to which they give rise. These two reports may present many subjects of doubt, for the solution of which the judge orders two or more practitioners to give their opinion of the previous reports; and at the same time furnishes them with all the documents in his possession capable of throwing light upon the enquiry. This is called a medico-legical consultation, in which, after a discussion and exposition of the facts, conclusions are formed either confirmatory or contradictory of those of the first examiners. Having obtained these documents, and collected the evidence of other witnesses, the judge draws up a faithful recital of the depositions both for accusation and acquittal, and submits it to the Chambre de conseil, composed of three Juges du tribunal de première instance, where it is determined whether the case shall be prosecuted. Having decided in the affirmative, this chamber also decrees whether the case shall be tried at once in the chamber of correctional police, or be carried into the court royal. If the affair is one of a very serious description, and consequently must be tried at the assizes, the Procureur général presents, either in person or by deputy, all his documents to the Chambre des mises en accusation, composed of counsellors of the court royal; and, as soon as this chamber has determined upon the further prosecution of the case, the accused person is brought before the court of assize. On the day of audience, all the medical practitioners appointed by the Commissaire de police and by the Juge d'instruction to make reports appear in succession as witnesses. The written reports being now considered in the light of mere materials of instruction, the witnesses testify *vivâ voce* of all that they related in the *procès-verbaux*. They answer interrogatories put by the jury, the judges, and the Procureur du roi; these questions being generally suggested by the depositions of previous witnesses, and in explanation of new facts that may have been elicited during the investigation. The trial is then closed.

If we compare this course of enquiry with that which in a similar case would be followed in our own courts, it can scarcely fail to strike us that fewer faults, both of omission and commission, are likely to be committed in the former; for the obvious reason, that the examination of the medical facts is consigned to the persons best qualified both for discovering and estimating them. We intrust to our coroner's inquest the charge of doing all that in the French prosecution precedes the submittal of the documents to the Chambre des mises en accusation, which answers to our grand jury. In other words, the coroner's inquest is responsible for as much as the two successive reporters, the persons engaged in the medico-legal consultation, the Procureur, the Juge d'instruction, and the Chambre de conseil. Without insisting upon the important consideration that, by passing through so many hands, the facts must be more nicely sifted, the medical information is of the fullest and most exact description. But how often have we seen the ends of justice defeated by the constitution of the coroner's inquest! Sometimes the medical witness is over-cautious or niggardly in his spontaneous statements; and, therefore, whether all that he knows shall be elicited depends upon the coroner and jury, who are probably altogether incapable of putting the proper interrogatories. The statement is given in non-professional language, (or, if otherwise, is apt to be treated with derision and contempt,) and consequently must be often scanty and equivocal. The early reporters in the French procedure are aware that their document is liable to be submitted to the critical examination of their brethren. They describe and interpret the facts according to the rules of the science, not in accommodation to the ignorance of other functionaries. A bare statement of facts may suggest a very erroneous idea to a mind unaccustomed to the survey of such facts, and may need a specific explanation on the part of the narrator; but it is a chance whether this explanation will be called for at the inquest, and medical witnesses may with some reason be wary of volunteering opinions, especially if they bear hard upon the accused. But, if the mental character of the witness is of a different kind, if he is hasty in inferring and rash in communicating, how will his declarations meet with the qualifications which they so much require, or his opinions be duly canvassed? If he chance to bear a reputation for sound sense and professional skill, his erroneous or prejudiced views—views, moreover, which he may hereafter find reason to modify,—will pass current, and an innocent person may lie in a dungeon till a trial can acquit him; or a culprit may altogether escape the vengeance of the law. It has often been agitated whether the function of a coroner should be executed by a medical man instead of a legal practitioner. We think that the arguments for both are nearly balanced, for the plain reason that the function is twofold. It is our earnest hope that no long period will elapse before the present duties of the office shall be so separated as not to be discharged by a single individual, and that there shall either be a medical as well as a legal coroner, or that the whole medical examination of the case shall be transacted by a body of men qualified for the purpose, either by their general professional attainments or by their particular education, like the *state-physicians* of Germany. Even if it were needless in ordinary cases, we hold such an alteration to be absolutely essential in cases involving medical *responsibility*. Our own observation of inquests held upon persons suspected to have died of *mala praxis* has taught us that the legitimate inferences from the facts actually deposed may entirely escape the coroner and the jury, for want of professional knowledge; so that at one time an atrocious charlatan may come out of the inquiry not only with impunity but even with eclat; and at another time a well-informed and humane practitioner may receive an undeserved but indelible blot upon his professional reputation.

Our author laments that legal medicine is not yet taught practically in France. He glances at the curriculum of study requisite for learning the sanatory branches of the profession, and then contrasts it with a course of medico-legal instruction. In the latter there is nothing practical, excepting perhaps what relates to toxicology. But of what does even this important part of the course consist? Of a succession of precipitates, which appear and disappear before the astonished eyes of the pupils, not one of whom is ever called upon to perform a chemical experiment, or to examine the body of a person drowned, hanged, or suffocated. "We have good reasons," he says, "for insisting that we require for medico-legal education something corresponding to the clinique of hospitals, in which pupils might find the same means of practical instruction as are afforded for medicine in general."* If these strictures are needed in France, they apply much more strongly to the British schools of medicine.

We pass over some forcible observations, in which M. Devergie censures the still insufficient attention paid to this department of medicine, and in which he urges the necessity of making it a separate object of study, and argues that a good physician or surgeon is not by necessity a good medical jurist. He regrets that there are no public functionaries in France corresponding to the state-physicians of Germany; for, notwithstanding the precautions taken for a thorough examination of the medical facts by separate sets of reporters, &c., the *first* observations, and therefore the most important, are often intrusted by the officers of police to practitioners very inadequately qualified for the duty. Hence the examination of the bodies is often incomplete, and facts of the greatest value to forensic medicine are utterly lost. He suggests the appointment of three orders of medical jurists: one to be attached to the court royal; another to the Tribunal de première instance; a third to be at the disposal of certain magistrates, such as mayors, Juges de paix, Commissaires de police, and Officiers de gendarmerie. We have often weighed the desirableness of such appointments for this country; but, while by no means blind to their advantages, (which indeed must strike every one who at all considers the subject,) we are disposed to think that very serious evils might result from them. One of the first effects, unless we are greatly mistaken, would be to render forensic medicine a matter of comparative indifference to all but those who look forward to an official exercise of this department of the profession. Now, it must necessarily happen that, however great the number and the activity of the medico-legal officers, the witnesses of many important facts must be persons engaged in ordinary practice. If a man is found apparently dying or dead, the first idea of the friends or spectators is not, whether he is to become the subject of a future judicial investigation, but whether any thing can be done towards his recovery; and the assistant sent for is not a medical jurist, but a practitioner of the art of healing, during whose attendance many phænomena may present themselves of the utmost importance in a medico-legal point of view, but which may escape his attention, from his want of information upon such subjects. This objection applies still more strongly to cases of illness, in the course of which no suspicion may have been excited in the mind of the attendant, but which would have afforded serious intimations to a toxicologist. Whether it might not be well to appoint officers to examine intricate cases, and to canvass the first reports, is a question upon which we have already ventured our

* Introduction, p. 11.

opinion. We only object to a plan which would seem to exempt the ordinary practitioner from the responsibility of exercising a medico-legal duty, by the appointment of a special functionary, who, in any given case, may not have an opportunity of observing the facts most essential to its elucidation.

We shall now select from the work before us a few of the special topics, both for the sake of the information which they contain, and to exemplify the manner in which the author has executed his task. To analyse the chapters seriatim would occupy far more space than can be afforded to the present article. We shall begin with the remarks upon Putrefaction, a subject which M. Devergie's office at the Morgue has well qualified him to discuss. He observes, that the descriptions given by Fourcroy and Thouret of putrefactive phænomena comprise four principal facts. (1.) The evolution of gas. (2.) Liquid putrefaction and disorganization of the soft parts. (3.) Arrest of putrefaction, and a transformation *en gras*, (adipocire.) (4.) Gradual destruction of the adipocire. The observations of MM. Orfila and Lesueur, on the contrary, assign to the gaseous and liquid putrefaction only an accidental occurrence. According to these distinguished authors, the first change is softening, the second desiccation, the third transformation *en gras*, the fourth and fifth stages are gradual destruction. The discrepancy between these observers Devergie attributes to the fact that most of the bodies examined by Orfila and Lesueur had been buried in winter, but those by Fourcroy and Thouret in summer. In some of Orfila's subjects interment had taken place in summer, but they were exhumed at a late period, when the appearances corresponded to those noticed by the other observers. The difference in short has relation principally to the earlier phases of decomposition. (T. i. p. 115.)

Gay Lussac was of opinion that putrefaction cannot occur in vacuo. Manners, Luischius, Fourcroy, and Guntz think differently. Guntz introduced his finger into a bell-glass filled with mercury, and then pricked it; blood having risen to the surface of the mercury, the vessel was submitted to a temperature gradually elevated from 15° to 30° (Reaumur.) The blood coagulated, and at the end of five days became liquid, dirty, and homogeneous, with bubbles of air upon the surface. Of all the gases, oxygen, according to Bæckmann and Hildenbrand, is most conducive to putrefaction, but its power is greatly increased by the presence of nitrogen, which, however, appears to exert no other action than that of separating the molecules of oxygen. An analogous fact is presented by hypophosphoric acid, which is formed very readily when its base is in contact with a mixture of oxygen and azote, but not at all when submitted to pure oxygen. Hildenbrand found that meat putrefied completely in eleven days when exposed to this gas in a state of purity. Unmixed azote appears to retard rather than to favour putrefaction; hence this process is slow in privies. Hydrogen, carbonic acid, and nitrous acid, are equally unfavorable. It is well known that electricity accelerates the process. Muscles subjected to a current of it are after a time deprived of their salts; the oxides going to the negative pole, the acids to the positive. In its ordinary action upon animal matter, it most probably alters the composition of the proximate principles; thus, in milk, it developes acetic acid. M. Matucci observes that portions of meat placed upon zinc plates remained fresh for a long time; the meat, being electro-negative, repelled the oxygen. Aqueous vapour is highly favorable. Gay Lussac found that he could retard putrefaction for a considerable period in a bell-glass containing at the bottom chloride of calcium. Devergie attributes to the solvent power of water its agency in promoting putrefaction. A stream delays the change, probably by removing the putrid particles first formed (which, if remaining in contact with the particles not decomposed, would hasten the process,) and at the same time guarding the substance from the contact of air. Chlorine retards by forming a white pearly compound almost imputrescible; deutoxide of nitrogen has a similar influence by absorbing oxygen; and sulphurous acid, by transforming the matter into a substance highly oxygenated.

The account given of Saponification contains nothing that need detain us. The reader will find a far more ample and exact discussion of the subject in the work of MM. Orfila and Lesueur. Neither shall we analyse or extract from the section on putrefaction of bodies underground, as it is avowedly a mere abstract of the researches of those indefatigable observers. Forty-seven pages are a literal transcript from the "Exhumations Juridiques," detailing the successive changes which the several organs and tissues undergo, and the modification of these changes by a great variety of circumstances.

We shall pass on to the author's observations upon putrefaction under water, a subject to which he has paid particular attention, and upon which he has more than once published in the Ann. d'Hygiène et de Méd. Lég. He arranges the phænomena of the putrefactive process in water under nine distinct heads: 1. *Green* putrefaction. This commences in the skin, and appears first over the sternum, gradually extending to the face, the neck, abdomen, shoulders, groins, arms, and legs, in the order in which these parts are named. (We may mention that, in atmospheric putrefaction, a different order is observed, being as follows: abdomen, groins, inferior region of the chest, neck, thighs, anterior part of the chest, legs, arms, and face.) It sometimes penetrates from the skin to the superficial muscles, but rarely to the deeper-seated, other phænomena having previously occurred. The green surface is often traversed by dark blue or black lines, caused by decomposition of blood in the vessels. The period at which this change takes place is about the third day in summer, and the twelfth or fifteenth in winter.

2. *Evolution of gas.* This occurs particularly in the stomach, intestines, lungs, and heart. In winter this change is much less marked than in summer. The pressure of the gas is often so great as to drive the blood back from the larger trunks into the smaller veins and capillaries, causing injection, imbibition, and general reddening of various tissues, more particularly in the cavities. These effects will of course be greater in proportion to the quantity of blood in the venous system; and hence we may derive hints as to the nature of the death, whether asphyxial or syncopal. The gas developed in the lungs may expel from the trachea the watery foam characteristic of death by submersion. Hence, in summer, we should be cautious of founding any positive opinion upon the absence of this sign. Chaussier attributed the transudation and the consequent reddening of the mucous membrane in drowned bodies to the peculiar fluidity of the blood. The period for the disengagement of gas is in summer about the fourth or sixth day, while in winter it may be delayed to the sixth or even the eighth week. It is obviously for this reason that bodies are observed to float much sooner in the former season than in the latter.

3. *Brown* putrefaction. This commences in the same parts as the green, but it is less disposed to spread, being for the most part overtaken by saponification. It seldom penetrates below the skin. Green and brown are the most usual tints, but they are sometimes interspersed with yellow, blue, and violet. The *brown* is attended with softening. The time of its appearance is about the first month in winter, and the tenth or twelfth day in summer.

4. *Putrilage,* which consists in liquefaction of the parts previously invaded by the green and brown putrefaction. It commences in the skin of the forehead, and extends in succession to that of the eyelids, nose, lips, clavicles, sternum, cartilages of the ribs, the abdomen, and groins. The destruction of continuity allows the escape of the gases previously evolved; these however have many other channels of egress, as the natural openings and the pores of the skin. The period is about the second or third month.

5. *Saponification.* The skin, not yet disorganized, becomes opaline, dense, and unctuous. The progress of liquefaction is arrested, and the volume of the parts increased. The muscles shrink and assume a rosy colour. The bones, where exposed, appear bright red. All the internal organs are more or less shrunken. The skin of the legs often becomes dense and of a yellow colour, presenting an appearance like parchment. The period is from the third to the fourth month,* being generally earlier in women, on account of their greater inclination to obesity.

* We must remind our readers that the period assigned by the author to the adipocirous process, does not refer to the formation of the substance under all circumstances. It is well known that Sir G. Gibbes's experiments led him to state that six weeks was a sufficient period for its production; but that gentleman operated upon detached portions of animal matter. M. Devergie's observations are confined to the change as it ensues in a body which has remained entire.—(Rev.)

6. *Desiccation.* This is often so considerable that the proper covering of some of the viscera, as the liver and the spleen, confine the putrid matter into which those organs are decomposed. It begins about the fourth month, but by this time the process of saponification has been penetrating the intermuscular cellular membrane. The muscles alone escape desiccation; they are red, glistening, moist, and yet not easily torn.

7. *Corrosions,* which have generally a granular surface. When caused by the solution of saponaceous matter, they are round and of small extent; when resulting from liquid putrefaction they are irregular and more extensive. The period is about the tenth week.

8. *Incrustations.* The substance of which these deposits are formed is a calcareous soap, resulting from a double decomposition between the sulphate and carbonate of lime derived from the water, and the margarate and oleate of ammonia which constitute adipocere. The skin is much thickened, while its density is increased to a degree that renders it even sonorous when percussed. The bulbs of the hair are singularly enlarged, presenting however a diversity of appearance according to the part of the body. Thus, upon the abdomen, they are disposed like small quills overlapping each other; on the thighs, they are less prominent, but more rounded; on the shoulders and back they are smaller, pyramidal, pointed, and arranged side by side. About the same time that these formations occur, saponification has invaded the muscles; those which are sheathed by aponeuroses being least transformed. The period is between four and five months. The brain is at this time entirely saponified, and the bones are very friable. The lungs are reduced to one-tenth of their natural volume; the cartilages of the trachea are separate; and the stomach and intestines are nearly destroyed.

9. *Destruction* of the soft parts generally. This is effected by the solution or detachment of the saponaceous matter, which leaves the bones almost bare. It occurs first upon the head, and afterwards upon the chest, abdomen, and extremities.

M. Devergie next adverts to the circumstances which modify the putrefactive process in water. We shall specify a few of these:— 1. The extent of the coverings of the body. The boots of men and the corsets of females are a great protection. The author states that he once met with the body of a female, in which, although it had been submersed five months and a half, the skin upon the trunk was quite natural; while upon the head it had undergone saponification, and upon the thighs and legs was covered with calcareous incrustations. 2. Stagnant water accelerates those stages of putrefaction entitled green, brown, and liquid. 3. The changes above detailed do not necessarily observe a certain order of sequence: they may be grouped, however, into two classes, mutually independent; the one comprising green, brown, liquid, and gaseous putrefaction; the other comprehending saponification, corrosion, desiccation, and incrustations. The former always affect the same parts, but they may be wanting in some cases; notwithstanding which the latter will occur, so that the former are not necessary antecedents. It must be mentioned, however, that the author has never witnessed a case of saponification throughout the body, without any liquid putrefaction. 4. An elevated temperature hastens the green, brown, liquid, and gaseous putrefaction, and consequently renders the body more disposed to float. In summer it is rare to meet with a body that has been more than three weeks in the water. 5. Whether the formation of gas is a constant phenomenon has not been ascertained. In winter, bodies often exhibit so slight a destruction of the skin, as to intimate that little or no liquefaction has taken place; a process which is known to be much favored by the production of gas. 6. The seasons of summer and winter produce at least a month's difference in the period at which the changes occur. 7. Saponification is often prevented in summer by the floating of the body. 8. Integrity of the skin is essential to the saponification of the whole body. 9. Saponification and desiccation occur more readily, *cæteris paribus,* in young subjects, and in those abounding in fat.

It was observed by M. Orfila, that putrefaction is less rapid in the water of privies. This fact is attributed by Devergie to the presence of a large quantity of ammonia, which retards the liquid putrefaction, but favours the production of adipocere. He arranges the different media with reference to their power of favouring putrefaction in the following order:*—Air, manure, the water of privies, stagnant water, running water, earth.

We cannot follow the author through his account of the changes which particular organs and tissues undergo during their immersion in water. He mentions the curious fact, that the pultaceous matter into which the brain is converted may find its way into the veins, and even reach the vena cava. We must find room, however, for the following table of the changes characteristic of particular periods. The reader will bear in mind that it is applicable only to bodies submersed during the winter months.

1. *From three to four days.* Loss of animal heat, rigidity, no contractility under the galvanic current; epidermis of the hands beginning to whiten.

2. *From four to eight days.* General flexibility. Colour of the skin natural, but the epidermis of the *palms* very white.

3. *From eight to twelve days.* Flaccidity. Epidermis on the *backs of the hands* beginning to whiten. Face softened, of a dull white colour, and in this respect differing from the rest of the skin.

4. *About fifteen days.* Face slightly soddened (*bouillie*), and here and there red. A greenish tint upon the middle of the sternum; the epidermis of the hands and feet entirely white, and beginning to wrinkle.

* It must be remembered that the author includes saponification in the putrefactive changes; otherwise, after the observation just made in the text, it would be an inconsistency to give the precedence to the water of privies.—(Rev.)

5. *About a month.* Face brownish-red, eyelids and lips green; a brown-red patch surrounded by green on the anterior part of the chest; the epidermis of the hands and feet white, swollen, and wrinkled, as if by poultices.

6. *About two months.* Face brownish and tumefied; hairs loose; epidermis of hands and feet nearly detached, nails still adherent.

7. *Two months and a half.* Epidermis and nails of the hands detached; also the epidermis of the feet, *but not the nails.* In females, redness of the subcutaneous cellular membrane of the neck; partial saponification of the cheeks and chin; superficial on the mammæ, groins, and anterior surface of the thigh.

8. *Three months and a half.* Destruction of part of the scalp, eyelids, and nose. Partial saponification of the face, neck, and groins. Corrosions. Epidermis of the hands and feet entirely removed, and the nails separated.

9. *Four months and a half.* Saponification of nearly the whole of the face, neck, groins, and anterior parts of the thighs; commencement of incrustations on the thighs, and of saponification of the anterior lobes of the brain. The skin of an opaline colour. Detachment of nearly the whole scalp. The skull beginning to be friable. (T. i. p. 227-228.)

This table was first published in the Annales d'Hygiène for 1830; and the author now informs us that he has found no reason to doubt its accuracy. The readers of the "Exhumations Juridiques" may remember that M. Devergie is made the object of some severe strictures for presuming to fix any dates to putrefactive processes, which must be influenced by a great variety of modifying circumstances. Devergie contents himself with observing that the correctness of his table has been tested, not only by himself, but also by others; that, in fact, the period at which persons had been drowned has been determined with considerable precision, by comparing the appearances which their bodies presented with the statements alluded to.

The author has not as yet been enabled to construct a similar tabular arrangement of the changes which take place during the summer; but he has made a few general observations worthy of attention. In the course of ten or twelve days in summer, putrefaction has been as far advanced as we generally find it after six weeks in winter. The face is puffed and of a brownish colour, the eyelids are swollen, and the lips voluminous. The whole body also is more or less swollen, the skin bears an opaline tint; a green spot may be observed upon the sternum, and the epidermis of the hands is quite soddened. Thus, there is a difference of at least twenty-two days in the time requisite for producing these appearances. The other observations we shall put into the form of a table.

| *In Summer.* | *In Winter.* |
|---|---|
| 5 — 8 hours | 3 — 4 days |
| 24 hours | 4 — 8 days |
| 48 hours | 8 — 12 days |
| 4 days. | 15 days. |

The changes in spring are somewhat intermediate to those observed in summer and in winter; but they are considerably affected by the previous season. If the winter has been very severe, the progress of putrefaction will be slower in the following spring. It is not, however, equally true that a hot summer renders putrefaction more rapid in the ensuing autumn; a fact which is accounted for by the well-known law, that water is more easily cooled than heated by an agent applied to its surface.

In calculating the period of submersion, we must always take into account the length of time during which the body has been exposed to the atmosphere after its removal from the water. Five hours only are sufficient to produce a very remarkable alteration of the appearances, more particularly in the earlier stages of putrefaction. Saponification for obvious reasons undergoes little or no change from this cause.

We have devoted a large proportion of our space to these observations, because we believe them to be comparatively novel to the majority of English readers. At first sight, they may appear superfluously minute, and tending to prove little more than that French investigators are not easily disheartened either by the laborious or the disgusting nature of their researches: but a very little consideration must be sufficient to convince us that any means of enabling us to determine with greater precision the period at which an individual died, must be important in a great variety of judicial enquiries, both civil and criminal.

We should have been glad to have made an abstract of the very excellent chapter upon medico-legal dissection, but content ourselves with recommending an attentive perusal of it.

The directions for conducting disinterments for forensic purposes are ample and explicit; but the author makes a rather sudden transition from this subject to that of Rape. He does not appear to have considered it needful to arrange his topics in any very nice order; but, if there can be said to be one principle of connexion more prevalent than another, it is physiological; and perhaps this is the most natural as well as the most convenient kind that he could have adopted, since it admits of his treating at the same time both criminal and civil questions involved in the same subject. Thus, Rape, Impotence, Pregnancy, Infanticide, Abortion, Viability, are connected, inasmuch as they all belong to the generative system; but the judicial questions arising out of them are extremely various, and, if discussed separately under the heads of civil and criminal, would occasion many needless repetitions of physiological observations.

In the chapter devoted to the signs of Pregnancy, we noticed one or two rather important deficiencies. The evidence derived from the state of the nipple is thus summarily discussed : " *Imbrowning of the nipples.* This character belongs to every case, but cannot be turned to account except when the person examined is young and in her first pregnancy, and when the lips and natural apertures are of a rosy colour, corresponding to whiteness of complexion; for, in brunettes, the nipple almost always presents a dark tint." The author entirely omits certain characters belonging to the part, of far greater consequence than mere colour. It is scarcely necessary to say that we allude to the turgescence of the organ, the development of the follicles, and that soft and almost humid condition of the skin within the areola which is easily distinguishable from the feel of the adjoining epidermis. These signs have been forcibly described by Rœderer, and insisted upon by Dr. Montgomery in his admirable monograph upon the evidence of pregnancy, published in the Cyclopædia of Practical Medicine. Dr. W. Hunter placed so much reliance upon the indications of the nipple and areola, that on one occasion, after having declared that the body of a female brought into the dissecting room contained an impregnated uterus, from his observation of the appearance in question, he was not moved from his opinion by the discovery of an inviolate hymen. An examination of the uterus verified his judgment. M. Devergie makes no mention of the projection of the umbilicus in the sixth month, a sign much esteemed by Dr. Gooch; and, when treating of the evidence obtained from dissection, does not enter at all into the much vexed and still unsettled question respecting *corpora lutea*. Upon the disproval of pregnancy he puts a query well worthy of consideration. The sound of the fœtal heart, the placental *bruit*, and *ballottement*, together, or even separately, may enable us to affirm with confidence the fact of impregnation; but will the absence of these and other signs justify us in pronouncing a negative with equal certainty? We fear not. It might be asserted that the proof of the gravid condition is wanting, but we could scarcely undertake absolutely to deny its existence.

The section upon Delivery contains nothing that particularly invites our analysis or comments. We shall pass on to an interesting article upon the Duration of Pregnancy, or, in other words, upon precocious and retarded births. The French law determines that, in questions of legitimacy, the 180th and 300th days shall be considered the extreme periods of gestation. We shall confine ourselves to the author's remarks upon the possibility of a protraction of this condition beyond the 280th day. Louis and Bouvart were the first to appeal to analogies in the lower animals, but, unfortunately, their facts which were taken from the writings of Buffon, were by no means correct. It is stated, upon this authority, that mares and she-asses always go eleven months, neither more nor less; cows nine months, deer eight months, sheep and goats five months, bitches two months, hares and rabbits one month. From facts of this nature it was inferred that the laws of nature are invariable, and consequently that a woman must bring forth in the commencement of the tenth month; in other words, that she cannot exceed the 280th day, as fixed by Hippocrates. Later observations, instituted by Tessier, have proved that naturalists, and Buffon in particular, had fallen into some errors upon this subject. Thus, out of 160 cows observed by Tessier, fourteen calved between the 241st and the 266th day, three on the 270th, fifty between the 270th and the 280th, sixty-eight between the 280th and the 290th, twenty on the 300th, and five on the 308th; so that there was a difference of sixty-seven days between the longest and shortest periods. Similar variations were noticed in the gestation of mares; the difference between the two extremes amounting to no less than eighty days. The most common term for cows was nine months and ten days; for mares, eleven months and ten days. It is worthy of note that, of the cows, five only passed the 300th day, (a fact to a certain degree consonant with the French law,) and it is probable that these animals are placed in circumstances far more favorable to protracted gestation than are women, in whom so many causes, both physical and moral, tend to hasten rather than to retard parturition. M. Devergie, after citing the evidence delivered in the celebrated Gardner peerage case, alludes to a case related by Velpeau of a woman in whom parturition commenced in the ninth month, and was then suspended for three weeks, that is, till the 310th day. It is now, we conceive, placed beyond all dispute that parturition may be deferred till some time after the 280th day. Even if actual facts had been wanting, the position might to a certain extent have been supported by mere *à priori* arguments. Why should pregnancy be more exempt from variation than other physiological conditions? Do the teeth appear at a definite period? The regular interval between the catamenial efforts is four weeks, but how often is this varied, sometimes by disease, sometimes by idiosyncrasy. The fortieth week is the natural period for the termination of pregnancy, and any departure from it is unnatural: but only in the sense that would apply to tardy or premature menstruation. Devergie entertains an opinion that the general law is that parturition shall begin at the tenth menstrual period, believing it to be more natural that the nisus should occur at one of the periods of uterine excitement than at any other period. He suggests that enquiries should be instituted as to the duration of pregnancy in women who habitually menstruate every three weeks. If it should appear that the time of gestation is shorter with them than with others, his notion of the existence of a direct relation between the effort of menstruation and that of parturition would receive no little confirmation. The arguments for and against protracted gestation are thus ably summed up. Those who deny the fact in question, urge that nature is unchangeable in her operations, that gestation never varies in the lower animals, that the fœtus, at a certain point of development, which it always attains in nine months, acts mechanically as a foreign body upon the uterine parietes, and thus provokes their expulsive contractions, just as the chick, when it has undergone development to such a degree that it can no longer procure a sufficient quantity of nutriment from the albumen of the egg, makes its way out of the shell. The advocates of the opposite opinion allege that naturalists have differed greatly in their statements of the length of gestation, not only in the same species, but even in the same variety; that some fix ten months for mares, others from eleven to twelve; and the same remark holds good with regard to cows and asses. Aristotle assigned two months to the bitch, Varro three, and Albertus Magnus from sixty-one to seventy-one days, or even three months. Pliny speaks of five months as the period for some races of sheep, but a later time for others. It is urged, moreover, that the human fœtus, like the young of the inferior classes, is not expelled from the womb till it has acquired a development adapted to extra-uterine existence; that disease and other causes may delay this development; and, consequently, that there is no reason for astonishment if parturition be sometimes retarded.

The foregoing discussion is followed by some remarks upon Superfœtation. After enumerating certain facts bearing upon this supposed condition, the author concludes that there are no well-authenticated examples of its occurrence, excepting under three circumstances. 1st. When the uterus is double; 2dly, in extra-uterine conception; 3dly, when the ovum has not reached the uterus. But, as the possibility of superfœtation in cases in which the uterus is single, and in which the product of one conception is supposed to have already entered that organ, has not yet been absolutely disproved, Devergie is of opinion that the virtue of a mother, and the legitimacy of her offspring, ought, *cæteris paribus*, to have the benefit of the doubt. There would not be much hesitation in an English court respecting such a case as the following, which our author states hypothetically in illustration of the difficult nature of the medico-legal enquiries that might arise out of an instance of superfœtation?

"A woman loses her husband in the ninth month of her pregnancy; she is delivered a few days after that event, and marries again in twenty days from the date of her accouchement. Eight months after her second marriage, she brings forth an infant *bien portant*. Now the question occurs: Does the child belong to the first husband by virtue of superfœtation, or to the second?"

It is almost difficult to treat such a case seriously; but, supposing the three preliminary events to have befallen according to Devergie's statement, we think that the paternity might be determined at once by ascertaining not only that the infant is *bien portant*, but also that it is *à termé*.

The author has done full justice to *Infanticide*, both by the extent and valuable nature of his observations. He commences with a consideration of the phænomena characteristic of the different periods of fœtal existence. It is impossible for us to follow him, even at a distance, through the details into which he has entered, with reference to this department of the subject; more especially as we discover no great discrepancy in the characters which he has collected, as compared with those presented by former writers, if we except his statements concerning the length of the fœtus at different periods, which differ considerably from those of Lecieux. We have in the following table arranged these statements in the manner best calculated to exhibit their difference.

*Length of the Fœtus.*

| | | Lecieux. | | Devergie. |
|---|---|---|---|---|
| Fifth month | . | 9½ inches | . | 6 to 7 inches. |
| Sixth — | . | 12 — | . | 9 to 10 — |
| Seventh — | . | 14 — | . | 11 to 12 — |
| Eighth — | . | 16 — | . | 13 to 15 — |
| Ninth — | . | 18 — | . | 16 to 18 — |

It is often important to ascertain *how long* a child has survived its birth. The author considers very carefully the signs indicative of the extent which the extra-uterine life has attained in its earlier periods. The following is a summary of the characters:

*After one day.* The withering of the cord has commenced, (in a direction from the summit towards the base.) The cord still united to the umbilical ring.

Foramen ovale . .<br>

Ductus arteriosus .<br>

Arteriæ umbilicales . } all open.<br>

Vena umbilicalis .<br>

Canalis venosus . .

*Two days.* Foramen ovale open in eighteen cases out of twenty-two.
Obliteration of ductus arteriosus beginning.
. . . umbilical arteries advanced.
Umbilical vein open.
Canalis venosus open.

*Three days.* Desiccation of the cord.
Foramen ovale sometimes closed.
Ductus arteriosus rarely quite obliterated.
Umbilical arteries very often obliterated.
. . . vein . . } still open.
Canalis venosus . . }

*Four days.* Separation of cord beginning.
Foramen ovale open in seventeen cases out of twenty-four.
Ductus arteriosus generally open,
. . . . . partly closed in seven,
. . . . . completely in three.
Umbilical arteries generally obliterated, but sometimes still open in the vicinity of the iliacs.
Umbilical vein . . } considerably contracted.
Canalis venosus . }

*Five days.* Separation of the cord generally complete.
Foramen ovale open in thirteen cases out of twenty-nine.

Ductus arteriosus open in half the cases.

Umbilical arteries . . .<br>. . . vein . . . .<br>Canalis venosus . . . } completely obliterated, (occasionally the vein is still open.)

Commencement of changes preparatory to the exfoliation of the epidermis.

*Eight days.* Separation of the cord complete; cicatrization commencing.

Foramen ovale open in five cases out of twenty.

Ductus arteriosus completely obliterated in half the cases.

Vessels closed.

*From the ninth to the eleventh day.* Cicatrization of the umbilicus often complete, but as often a mucous exudation remains until the complete obliteration of the vessels, and may continue till the twenty-fifth day, so that the skin may not be healed till a period still later.

*From the twentieth to the twenty-sixth day.* Complete exfoliation of the epidermis, except on the hands and feet, in which places it may not take place until the fortieth day. (T. i. p. 520.)

We think our readers will allow the following passage to be a very graphic description of the appearances presented by the body of a fœtus which had begun to putrefy in the uterus of the mother: it presents some striking differences from the phænomena of putrefaction induced by exposure to the atmosphere.

"This state can never be mistaken by a person who has met with it once or twice only; but, though easy to be recognized, it is somewhat difficult to be described. Let us suppose the little corpse to be extended upon a table. The first appearance that strikes us is an extreme flaccidity of all the soft parts; the head, place it in what position you will, is flattened by mere gravity; the forms of the ribs are perceptible through the integuments; the front of the chest is depressed; the abdominal parietes sink in about the umbilicus, but they bulge out in the iliac regions; the limbs have the same flabby appearance and feel as the other parts. The skin has a peculiar brownish-red colour, without any tinge of green; sometimes confined to the abdomen when the body has not been retained in the uterus for any great length of time after death, but often quite distinct upon the chest, neck, head, and extremities. It is quite different from the brown hue which succeeds the green in ordinary putrefaction, being of a brighter character. The cord is no longer contorted; it is cylindrical, fleshy, flabby, reddish, and infiltrated with a brownish liquid. The epidermis is more or less detached; but, even when still in contact with the skin, it readily peels off, leaving the cutis bare and moistened with a sort of mucous fluid: the colour of the skin under such circumstances is bright red. The epidermis of the feet and hands is white, thick, and wrinkled, as if from poulticing. A reddish serosity pervades the subcutaneous cellular membrane, as well as that which separates the muscles, and sometimes even the muscular tissue itself. The bones of the head are so loose as to be very easily moved upon each other, and the pericranium is easily detached. The cellular tissue of the scalp is infiltrated with a serosity which Orfila has very fitly compared to gooseberry jelly." (T. i. p. 527.)

The following question was once proposed to Devergie by an advocate-general, in a court of assize: "In forensic medicine, to live is to breathe, and consequently, as a medical jurist, you can only infer the life of an infant from the evidence of respiration; but may not a jury derive their proofs of life from other sources than respiration?" Devergie thought proper to answer that the solution of the question did not belong to him, because the law confines itself to asking whether the jury are convinced, without enquiring the grounds of their conviction; and therefore the question is resolved by the mere text of the law. But is a physician authorised in saying that an infant has lived though it has not respired? This question was invested with great interest in the celebrated cause of Fish *v.* Palmer, tried in this country many years ago. But, though the question cannot again be connected with the law of *tenant to the courtesy,* since the latter has been recently annulled, we may have to consider it in relation to infanticide. Our author properly observes, that a period amply sufficient for the operation of causes destructive to the infant often intervenes between birth and respiration. Thus, in some cases, death is caused by the gorged condition of the lungs; in others, by the syncope of the mother during parturition; and, in others, by the anæmia consequent upon maternal hemorrhage. It is possible that a woman might choose to be delivered in a bath, and thus to prevent the respiration of her infant.

"The proofs of life," says Devergie, "in all such instances, must be derived from certain lesions, produced by wounds or other kinds of violence. In some cases these injuries are so very distinct, that it is difficult not to suppose that they were occasioned during life; and, though such cases may be very rare, we ought to keep in mind the possibility of meeting with them. Let us suppose it proved that an infant born at the full time has not respired, but that the body presents a large ecchymosis of the scalp, with *coagulation* of the effused blood, a fracture of one of the cranial bones, a laceration of the dura mater opposite to the fracture, an extravasation of blood upon the surface of the brain, several lacerations of the liver, with effusion of blood into the cavity of the peritoneum, partly liquid and partly coagulated;—I confess that, in such a case, I should be prone to entertain a strong suspicion, if not to consider it an absolute certainty, that the infant was alive at the time the violence was committed." (T. i. p. 531.)

We conceive that the author has allowed himself to be unnecessarily puzzled by the equivocal signification of the term *life.* In the case supposed, there could be but little doubt that the injuries were inflicted during life; but what life? Fœtal or extra-uterine? We have no means of distinguishing the one from the other, but by the occurrence or non-occurrence of respiration. The case in question, then, ought to be considered in strictness with reference, not to infanticide, but to fœticide,—not to the *extinction,* but to the *prevention,* of extra-uterine existence. All fatal injuries committed upon a fœtus which has not respired, whether the lesion has been the effect of criminal abortion, or of artificial delivery, or of violence inflicted either during labour or after labour, must, in a physiological sense, belong to fœticide. A criminal court, sitting upon M. Devergie's hypothetical case, would have to satisfy itself that the fœtus would have respired but for the injuries sustained just after birth; and, this point having been established, the crime would most probably be treated in the same light as if it had been committed during parturition. We are not aware that any cases have come before the English courts illustrative of this subject.

The possibility of uterine respiration is a topic well worthy of consideration in a discussion of infanticide, since it tends to throw some doubt over the usual proofs of extra-uterine life. The fact of *vagitus uterinus* has been fully established, but some have found reason for questioning whether it necessarily involves the fact of respiration. M. Billard, who first directed attention to the two different sounds composing the cry of an infant, (viz. one produced during expiration, and the other during inspiration,) suggested that a quantity of air sufficient to produce the latter sound might traverse the glottis, and yet not reach the air-cells, and that this feeble penetration of air, though adequate to the production of a uterine cry, would not effect such changes in the lungs as occur in respiration after birth. Devergie, while admitting the distinction between the two sounds, is unable to conceive how the inspiratory cry *(reprise)* could take place, excepting after a full expulsion of air from the lungs, and consequently attended with a more ample instead of a slight respiration. Reasoning upon this subject must be purely analogical, since there are no experimental observations to guide us; that is to say, no examination has been made of the lungs of infants which had presented the phenomenon in question, and died during parturition. The probabilities appear to us to be almost equally balanced, since notwithstanding, in the ordinary cry, the *reprise* succeeds the expiration, yet, in certain kinds of spasm of the glottis, sound is produced only when air enters the passage, as in the crowing inspiration of infants. But, in whatever manner this question may be determined, we doubt whether much practical difficulty is likely to ensue from a belief in the possibility of uterine respiration. The evidence of respiration is of little value in a question of infanticide, unless it is proved that the respiration was pretty complete; for it often happens that children, though born alive, die after a few feeble inspirations, and, consequently, in these cases there may be proofs of respiration in a limited portion of the lungs. The vestiges, therefore, of such an amount of respiration as could take place in the uterus would form but a very small part of the evidence that death had occurred by other than natural causes. Besides, it must never be forgotten that, in a charge of infanticide, the practitioner has not only to prove that the child has lived and died but also that it has been killed.

The signs of respiration are treated in four classes: 1st, those derived from the thoracic parietes; 2dly, from the state of the lungs; 3dly, from the organs of circulation; 4thly, from the organs of digestion. The remarks upon the first of these classes present nothing which may not be found in other works. Under the second head the author describes a condition of the pulmonary substance which, with regard to its volume, might be mistaken for the development produced by respiration. He claims to himself the merit of having been the first to notice the lesion, and designates it as *œdème pulmonaire* or *endurcissement lardaciforme:* the consistence, he observes, is intermediate to the induration presented by the lardaceous form of scirrhus and the ordinary ramollissement of the lungs of new-born infants. We are surprised to find M. Devergie asserting that the altered colour of the lungs induced by respiration is not imitated by artificial inflation. Orfila states that a scarlet injection may result from the latter cause;* and the late Mr. Jennings, of Leamington, in a valuable memoir† upon Insufflation of the Lungs of Still-born Infants, testifies to the same fact in the most unequivocal terms.

The remarks upon Ploucquet's celebrated test are valuable, inasmuch as they must tend in some measure to repair the faith of the profession, which was seriously shaken by the researches of Chaussier in Paris, and of Schmidt in Vienna. It will be remembered that Ploucquet, after an examination of four subjects, arrived at the conclusion that the weight of the lungs of an infant which has never breathed is to that of the whole body as 1 : 70, while, in one which *has* respired, it is as 1 : 35; the increased weight being occasioned by the increased quantity of blood in the pulmonary parenchyma. But Chaussier and Schmidt, from a very extended series of observations, (the former having examined 400 subjects, the latter 101,) inferred that the difference is by no means so great as that which Ploucquet stated. Thus, Chaussier gives the relative weights of the lungs of the infant which has, and of that which has not respired, as about 1 : 49 in the former, and 1 : 39 in the latter. Schmidt gives the proportional numbers as 1 : 52 and 1 : 42.‡ Our author offers some good reasons for entertaining a little scepticism respecting the conclusions drawn from Chaussier's tables, many of which reasons would most probably apply to the statements of other observers. His objections are, that he has detected inaccuracies in several of the calculations; that the cases examined range from the sixth month of the fœtal life to the second year of the extra-uterine; that, in a considerable number of the subjects, the lungs were putrefied, wholly or in part, (a circumstance which could

* Leçons de Méd. Lég.

† Transactions of the Provincial Med. and Surg. Association, vol. ii.

‡ Hartman, a Danish physician, not referred to by Devergie, states the numbers as 1 : 59 and 1 : 48. See Dr. Arrowsmith's article "Infanticide," in the Cyclopædia of Practical Medicine.

not fail to modify very materially the results of the investigation, since the escape of blood and other fluids in putrefaction must necessarily diminish the weight of the lungs;) that, in several instances, the lungs were diseased, and sank in water, notwithstanding they belonged to infants which had survived ten, fifteen, or twenty days; and, lastly, that, in some of the cases, the lungs were taken from monsters, in which there was a deficiency of the proper balance between the development of the lungs and that of the rest of the body. These considerations are certainly quite sufficient to impair our confidence in M. Chaussier's inferences. In order that he might arrive at more correct conclusions, M. Devergie expunged from the tables in question all the cases liable to the above objections, and selected 200 as most likely to afford him accurate data. The differences in the proportions occasioned by the period of life, both intra-uterine and extra-uterine, are very striking. At the ninth month, in the infant which has not respired, the weight of the lungs is 1-60th of that of the body; after respiration, for a period varying from a few minutes to twenty-four hours, it is 1-45th, while Ploucquet made it 1-35th. The longer the period of respiration, the greater is the difference. After three or four days, the increase of weight by respiration is nearly one-half; but there is little utility in this observation, since the crime of infanticide is seldom committed at so late a period. It is worthy of remark, and favorable to Ploucquet's test, that, upon comparing the children still-born at the ninth month with those born alive at the same period, it appears that, in only two out of thirty-three, was the proportional weight of the lung greater in the former than in the latter. Moreover, in one-third of the still-born cases at this age, the weight of the lungs was no more than 1-70th of that of the body. At the eighth month, the difference in the weight of the lungs is more decided than at the ninth: thus, in those which have respired, it is 1 : 37; and in those which have not, 1 : 63. At the seventh and sixth months, the difference is too slight to render the method of Ploucquet at all serviceable. For the accuracy of these statements we must refer the reader to the author's tables. We have said enough, however, to shew that his researches encourage us to expect, at all events, much valuable collateral evidence from the test under discussion.

M. Orfila thought it desirable, in consequence of the variable results from Ploucquet's method, to endeavour to procure some more definite conclusions from the proportion between the lungs and the heart, before and after respiration, hoping to find less variation in the weight of the heart than of the whole body; but the experiments instituted with this object in view yielded nothing satisfactory.

The remarks upon the Hydrostatic Test are full, practical, and for the most part unexceptionable. Our readers are doubtless familiar with the objection founded upon the fact, that floating of the lungs may be caused by putrefaction, and the consequent evolution of gas; but there are several circumstances which considerably modify the application of this fact. Thus, it has been proved by repeated observations that, although putrefactive emphysema may occur in the lungs of a still-born fœtus after exposure to the air, it only occurs at a very late period, since these organs are almost the last to undergo the change in question. The experiments of Orfila prove that, if the body has remained in water, many months may elapse before the lungs become putrid: he states, indeed, that the integuments of the throat must have been previously destroyed by decomposition, and the pulmonary substance exposed to actual contact with water. The practical use of the last observation is well guarded by Devergie. The subjects of Orfila's experiments were bodies which had been kept in water during his researches; but Devergie examined the bodies of two infants which had been thrown into the Seine eight or nine days previously to his inspection of them, and he found a decided gaseous putrefaction in the lungs. The apparent inconsistency of this fact with the experiments of Orfila is removed, when we learn that the bodies examined by our author had been taken out of the river between twenty and thirty hours before the chest was opened, and during that period were exposed to the atmosphere.

It has been long observed that, if the body of an adult is removed from the water after several days of immersion, at a time when the temperature of the atmosphere is above 60°, the disengagement of gas commences almost immediately, not only in the external but also in the internal organs. The quantity of gas disengaged is sometimes so considerable as to alter the position of the limbs, and even of the whole frame; so that it is the practice at the Morgue to fasten the bodies down, in order to prevent them from falling from the tables. Sometimes these movements have been such as to induce strangers to report to the keeper of the establishment that some of the corpses had given signs of life. Now, that which occurs in the body of the adult under such circumstances is occasioned with no less rapidity in the fœtus, and, as it generally happens that the medico-legal inspection is not performed till several hours after the removal of the body from the water, the putrefactive emphysema will have advanced to a great extent. These facts, while they afford valuable proof of the possibility of the floating of the lungs by mere putrefaction, throw little or no difficulty in the way of the medical jurist, since he can easily distinguish this condition from that which depends upon the introduction of air into the cells. If unable to recognize the putrefactive emphysema by the presence of bullæ in the cellular tissue which separates the lobules, and by the absence of crepitation, he may resolve his doubts by observing the effects of compression, which will cause the pulmonary substance to sink immediately. To discriminate the effects of respiration from those of *insufflation* is far less easy; for, notwithstanding the penetration of air by artificial inflation is far less complete and extensive than in the natural respiratory process, it may produce nearly all the signs of the latter, —viz. elevation of the thorax, descent of the diaphragm, augmentation of the volume of the lungs, expansion of the air-cells, and (according to Devergie and many other authorities,) floating of cut portions of the lungs, *even after forcible compression.* He says that a practised eye may, in the case of insufflation, discover a difference in the general paleness of the pulmonary tissue, and in the absence of the injected capillaries observable after respiration; but he allows that these characteristics alone are not to be relied upon. The test of Ploucquet must be resorted to; but this also will not receive our entire confidence, for reasons already adduced. Unable to get completely rid of the difficulty, our author points out the extreme improbability that insufflation should have been practised in a case of suspected infanticide; for, supposing that some malicious individual, bent upon imputing the crime to the unfortunate mother, (a case which has never occurred,) should have inflated the lungs of the still-born infant, it is necessary to suppose also that the person is possessed of some professional knowledge, particularly of the fallacy to which the hydrostatic test is subjected by the procedure in question; and that chance favoured the plotter so far that a still-born infant is brought forth, that the mother has concealed her pregnancy and delivery, and that collateral evidence bears unfavorably upon her. Even supposing all these circumstances to conspire against the victim of malice, it remains to be proved not only that there are such signs of respiration as may be imitated by artificial introduction of air, but also that there is a *corpus delicti;* that is, that there is evidence of death by unnatural causes.

The latter observation would apply with equal force to another far-fetched supposition, to wit, that the mother, influenced by strong affection, may have resorted to artificial means for animating her infant; means which are afterwards to criminate her. "In imagining such a case," says Devergie, "it is necessary to leave out of account all the collateral proofs requisite for bringing home an accusation, and to forget that a murderess would strike her victim in secresy; while a mother, who could lavish such pains upon her infant at such a time, would act with perfect openness."

We should be guilty of a considerable omission were we to neglect, upon the present occasion, to draw the reader's attention to the experiments of the late Mr. Jennings, which have been already alluded to, and of which M. Devergie is evidently ignorant. Mr. Jennings satisfied himself, by repeated trials, that the pulmonary tissue may be made to sink after insufflation, by an extreme degree of compression; but that no degree of it can produce the same results upon lungs which have respired. His mode of operating was to knead the tissue in the palm of the hand, till it was reduced to a kind of paste.

It has been objected to the hydrostatic test, that the lungs may sink notwithstanding the child has respired, in consequence of the specific gravity having been increased by a morbid process of condensation, or by accumulation of blood in the parenchyma; but the researches of Orfila and Billard render it probable that floating may always be effected by expressing the fluids from the tissue. The volume and colour of the fœtal lung, the form of the lobules, and the acute angles at the borders of the lobes, will nearly always be sufficient to distinguish it from the state of morbid condensation sometimes found in the lungs of infants born alive: or, should we remain in doubt upon this subject, the accused cannot suffer from our uncertainty unless there are proofs of a violent death, not to mention that the existence of such a disease as would cause the lungs to sink is in itself a strong presumption that the death was natural.

We must pass over the remarks upon the tests proposed respectively by Daniel and Bernt; and we regret that we have not space for the author's very excellent summary of the signs affirmative and negative of respiration.

The next question that presents itself is, how long did the infant live? The answer will in many cases be assisted by the appearances already detailed. These, however, will throw but little light upon the question whether life continued only a few seconds or was prolonged for several hours; nor can we be guided entirely by the extent over which the air-cells are expanded, since this must vary extremely in different subjects.

Upon the means of judging how long a time has elapsed since death, no very exact researches have as yet been prosecuted. Orfila, with the assistance of Gerdy and Hennelle, observed the progress of putrefaction in portions of the fœtus; but this process is very different from the decomposition of an entire body, and consequently the researches of these gentlemen are of little avail, except in enabling us to form an opinion upon a case in which the body has been cut up into pieces previously to its concealment. (T. i., p. 594.)

The causes of natural death, during the course of labour or soon after birth, are treated with sufficient accuracy and minuteness, but we meet with little or nothing peculiar to the author. Under the head of death by violence, he alludes to a mode of suffocation more common in France than in this country, namely, the insertion of a plug in the air-passages. In the chapter upon Medico-legal Dissection, he describes the difference presented in the appearances according as the plug had been introduced during life or after death.

"The following," he observes, "is what I have noticed in two cases of suffocation by this method. The plugs (*tampons*) are generally made of folds of linen tightly bound together. As the cavity of the mouth and pharynx lessens towards the œsophagus, the degree of pressure occasioned by the plug must be greater in the lower parts. From this inequality of pressure there results a peculiar condition of the palate and pharynx in different parts, and a difference in colour at the two extremities of the plug. The mucous membrane is thin, white, and free from injection in the lower part, where the pressure is greatest; while, in the upper and anterior part, it is red or violet-coloured, turgescent, and thickened; appearances easily explicable by the obstruction which the plug offers to the circulation in the part. The plug itself is white, and moistened at the extremity corresponding to the greatest pressure, and sometimes dry in the inner folds; while the part which was free in the mouth is stained vermilion by the bloody exudation, and moistened through its whole thickness. These appearances can only be produced in a living infant; and, if they do not afford irrefragable proof of suffocation, they at least convey a very strong presumption of criminal intention." (T. i., p. 294.)

It is obviously a matter of no slight importance to be able to determine the degree of reliance which may be placed upon marks of contusion, with reference to injuries during labour. One of the most unsettled questions upon this subject respects the evidence of strangulation by twisting of the umbilical cord round the neck. Obstetricians are well aware that this accident is by no means an infrequent cause of death, especially in cases of tedious labour. If the infant has not breathed, the enquiry into this cause of death possesses no great interest in a medico-legal point of view; but if, on the contrary, after respiration has occurred, either in the vagina or (which more frequently happens) between the expulsion of the head and that of the trunk, death is occasioned by the pressure of the cord, either in the way of apoplexy or of asphyxia, it becomes interesting to ascertain whether the body will present appearances characteristic of the cause of death; such, for instance, as will enable us to distinguish them from the effects of artificial strangulation. Authors are not as yet agreed as to whether ecchymosis is ever occasioned by the pressure of the umbilical cord. Klein disputes the probability of such a result, upon the ground that, although he has examined a considerable number of infants who came into the world with the cord twisted round the neck, he has never detected any traces of it, either in the form of actual ecchymosis or of any impression whatever. He alludes to the fact that children born with the aid of forceps or of loops applied to the limbs, are often free from the least sign of cutaneous ecchymosis; and urges how improbable it is that this lesion should be produced in the infant by the pressure of such a substance as the umbilical cord, when it is so rarely found in the bodies of adults who have died by hanging. Devergie fully coincides with Klein, both as to the non-occurrence of ecchymosis from the alleged cause in the infant, and the extreme rarity of it in cases of death by strangulation.

Upon the latter point we shall have more to say presently. In the mean time we may observe, that our author appears to be ignorant of some well-attested cases related by German physicians, in which the necks of infants strangulated by the cord bore evident marks of the impression. M. Taufflicl has published a collection of these cases in the Annales d'Hygiène, for October, 1835; most probably after our author's remarks had gone to the press. We think it right to say, that M. Devergie uses the term ecchymosis in its most rigorous sense, and that, in the cases in question, he might fairly deny its existence, notwithstanding there were other indications of compression of this part; such as a red, or brownish discoloration of the skin within the furrow. The cases, however, are quite at variance with Klein's assertion respecting the absence of any impression whatever. There is considerable foundation for our author's scepticism as to the possibility of mistaking the impression left by the neck of the uterus for the effect of a noose. Upon this point M. Velpeau observes, that in the supposed case the head of the infant must be either within the vagina, under which circumstances the neck of the uterus cannot operate upon the neck of the child; or the head must have passed the os externum, and then the cervix is too far back, and too much distended by the shoulders, to exert any pressure upon the neck of the infant. But, though the cervix may be otherwise engaged, we are far from feeling convinced that the os uteri may not press the neck, and that very tightly, after the birth of the head; whether with sufficient force to leave a circular impression, is far more questionable.

Devergie closes the consideration of this subject by reciting a case which was published in the Thirteenth Vol. of the Annales d'Hygiène, by M. Marc, who was engaged in the investigation. Though it is impossible for us to go into the details, we may be able to give such an outline as will exhibit the principal difficulties of the case.

The body examined was that of an infant at full term. The cord had been cut within two lines of the navel; the neck presented a circular furrow, a line and a half in breadth, in which there were two red bands, extending from before backwards in a horizontal direction to the nucha, where they were interrupted by portions of skin of a natural colour; the distance between the two streaks was half a line, and in this interval there was no discoloration. On each side of the neck there were irregular ecchymoses crossing the furrow; the nucha presented a longitudinal impression of deeper colour, four lines in length, and one quarter in breadth; the skin was frayed in this part, and there was extravasation in the subcutaneous cellular membrane. There were ecchymoses of the scalp, fractures of the parietal and occipital bones, and extravasations of blood in the convolutions of the brain. The lungs presented unequivocal signs of respiration. Upon these and other facts, M. Marc and his associates reported that it was difficult to assign the death of the infant to strangulation by the cord, because the furrow did not correspond to the ordinary volume of the cord, and because the red bands were separated by an interval of skin perfectly pale; and, upon the supposition that there had been two turns of the cord, the narrowness of the interval would not admit of explanation. The fact of respiration on the one hand, and on the other the absence of any sign of asphyxia, were inconsistent with this kind of death. The injuries of the head were amply sufficient to account for death; but how had they been produced? The mother declared that she was delivered while standing, that her infant dropped upon the floor, that she took it up, but that she fainted, and that it then fell from her arms. The woman was of low stature, and the distance between the os externum and the ground was too short for the first fall of the infant to have produced the lesions in the head; but they were more likely to have been caused by the fall from the arms. Such was the substance of the first report. A subsequent report stated that an examination of the pelvis of the mother had proved it to be large, and that the funis attached to the placenta was thirty-two inches in length; circumstances which explained both the sudden expulsion of the child, and its falling to the ground without any check. The extract from the requisition to the *procureur du roi* stated that the mother was delivered when alone in her chamber, that her child fell upon the ground, and cried; that she took it up, and opened the door to ask her younger sister for a pair of scissors; that, having obtained them, she closed the door, went to her bed, and then lost her consciousness, at which time her infant must have fallen a second time; and that, when she recovered her senses, she cut the funis, and placed the child upon the bed, but it was no longer living.

M. Marc characterizes this case as one of a most obscure description. He thinks it possible that the funis may have twice encircled the neck, though the furrow did not correspond to the *ordinary* size of the cord. Strangely enough, he omits to mention what was the circumference of the cord in this particular case. Strangulation, however, he considers to have been quite out of the question; and he attributes the death to the injuries of the head, the severity of which he explains by supposing that the mother might have thrown her infant down in the convulsive motions of syncope. With reference to the section of the cord being so near the umbilicus, he only observes that there were no indications of umbilical hemorrhage.

Devergie criticises the conclusions of the reporters, and expresses his own opinion in favour of the charge of infanticide: 1st, because the funis could not possibly have produced the fraying (*éraillement*) of the epidermis, the ecchymosis of the skin and cellular membrane, and so narrow an impression; and, 2dly, because the alleged fall of the infant from the horizontal position in the arms of the mother would not account for so severe an injury of the bones of the cranium. With respect to the suggestion that the child might have been jerked from the arms in the convulsive movements of the mother, he thinks it far more probable that the child should have fallen during that muscular relaxation which accompanies the first stage of syncope; but, even if convulsions had come on at once, what kind of convulsion, he asks, would make the arms act in such concert as to perform those movements of flexion and extension requisite for projecting such a body as an infant, and in such a manner that the head should fall perpendicularly upon the ground? These strictures are, we think, not hypercritical. The author might have pointed out the improbability that the extravasation upon the sides of the neck should have been occasioned by the funis. We regret that the circumference of the cord was not stated, because we have met with cases in which this appendage was sufficiently small to have left a furrow no broader than that described in the report; but, after weighing in our minds all the facts detailed in the documents referred to, we feel inclined to suspect that attempts were made to strangle the infant by some slight band (a string or ribbon for instance,) but that its destruction was completed by blows upon the head.

Interesting and important as are all the enquiries connected with Infanticide, we must leave many of them untouched. It appeared to us that a better idea of the author's capabilities and resources would be conveyed to the reader by following him pretty closely through one or two discussions, than by giving a hasty digest of so large a number of topics as must necessarily be treated in a systematic work upon Forensic Medicine.

We shall conclude our notice of M. Devergie with some remarks upon Death by Hanging; not that this subject immediately follows Infanticide in his work, but that some of the enquiries comprised in it are intimately connected with the question which has just engaged our attention. He does not trouble himself to present an historical survey of the various opinions which have been promulgated respecting the cause of death in cases of suspension. Persons moderately familiar with the literature of juridical medicine must be well aware that great authorities may be quoted respectively in favour of the doctrines which assign the death either to apoplexy or to asphyxia. There can be little doubt that the diversity of opinion among authors has arisen from the different nature of the cases which fell under their observation. The recent investigations of some German practitioners have, in our opinion, rendered it highly probable that the mode in which the noose was applied materially influences the production of the one state or the other. Professor Remer, of Breslau, (whose researches will be found in the Fourth Vol. of the Annales d'Hygiène,) collected 102 cases, nine of which presented signs of pure apoplexy, six of pure asphyxia, sixty-eight of the operation of both these causes, some of them shewing an equality of operation, others a predominance either of apoplexy or of asphyxia; in nineteen, the proximate cause of death was not ascertained. Dr. Fleichmann* asserts that, when the cord takes a horizontal direction, comprehending the larynx or passing immediately below it, or even when it passes between the os hyoides and the chin, and yet in its course to the nucha escapes the mastoid process, there will be discovered signs of apoplexy, or of the combined effects of apoplexy and asphyxia. But if, on the contrary, the cord, being situated between the thyroid cartilage and the os hyoides, or even in front of the latter, passes over the angles of the lower jaw and the mastoid processes, there will not be found any marks of apoplexy. The cause of the difference in these cases is that, in the former, the large vessels of the neck are compressed, but not in the latter, since the cord, though pressing upon the aperture of the windpipe, is prevented from interfering with the return of the blood from the head, by the angles of the jaw, and by the mastoid processes. In some few cases, however, notwithstanding the cord passes below the larynx and encircles the neck, there are no marks of apoplexy. Fleichmann imagines that, in such cases, death is hastened by some injury of the nerves of the neck. They correspond to those which Remer attributes to nervous apoplexy.

The author devotes a proper share of his attention to the necroscopic signs of death by strangulation. He offers some rational doubts respecting the common statement, that the position of the tongue relatively to the teeth depends upon the situation of the cord. His reasons are as follows:

1stly. I have found the tongue projected in a body which bore unequivocal signs of death by drowning, and upon which there was no impression of a cord. 2dly. I have met with two cases in which the tongue was projected, notwithstanding the cord was applied above the os hyoides. 3dly. I have produced the same effect in the dead body by fixing the cord in this situation."

Fleichmann avers that the position of the tongue is affected by the kind of death: thus, if the death was slow and distressing, the tongue will be found protruded. Devergie has met with great variations of the sign, and is disposed to consider it as dependent rather upon nervous than upon mechanical agency. (T. ii. p. 385.)

The appearance of the neck is far more deserving of attention than that of the tongue. Before considering Devergie's remarks upon this subject, we may as well state that a good deal of confusion prevails in medico-legal works as to the use of the term *ecchymosis,* particularly in connexion with the mark left by the cord upon the neck. As used by some writers, this word signifies the peculiar condition of the skin within the furrow; by others, the extravasation in the subcutaneous cellular membrane; and by others, again, the discoloration of the lips of the furrow. It

* See Annales d'Hygiène, t. viii. p. 424.

is only by remembering these differences of signification that we can account for the fact that Remer declares that nine-tenths of his cases presented ecchymosis; while Klein relates fifteen cases, and Esquirol twelve, in none of which was this lesion discovered. Devergie found it wanting in twenty-five cases, and Fleichmann in five out of six. But authors are well agreed that a peculiar discoloration of the skin under the cord, a brownish yellow not unlike the appearance of parchment, or of a blistered surface after death, is nearly always found; and we have little doubt that this condition has often passed with superficial observers for the dirty yellow colour which is left by an ecchymosis. A degree of pressure inadequate to the production of ecchymosis is quite sufficient to occasion the appearance alluded to. It was found in nearly the whole of that curious collection of cases which M. Marc appended to his medico-legal discourse upon the death of the Prince de Condé. In these cases the noose was tightened partly by voluntary efforts, and partly by the weight of the body, but without the additional force produced by a fall, as the lower extremities were in all the cases within reach of the ground. The instruments made use of were such articles as handkerchiefs, twisted shirts, &c. It is to be wished that we could admit M. Marc's statement that the parchment-like change in the neck cannot be effected after death; but M. Devergie has demonstrated by experimental researches that it may be cadaveric, and consequently that its value as a sign, when taken singly, is inconsiderable. The production of this state of the skin Devergie limits to two circumstances: 1st, when the cord has been applied with violence; 2dly, when it has been removed soon after death, and the skin has been exposed to the atmosphere, or even when the cord has remained in contact with the skin, provided several days have elapsed. He regards the alteration as the result of a purely physical process of desiccation; the fluids being driven out by pressure, the laminæ of the dermis being brought closer together, and rapid evaporation of the remaining moisture being favoured by exposure. In this process, the lips of the furrow often assume a violet colour, in consequence of the blood being driven into them from the part under pressure; an appearance which Esquirol and Devergie have produced in the dead body.

We do not consider exposure to the air so necessary to the desiccation as the author would intimate; for we have found the skin in a semicorneous state even an hour after death, and when the cord had not been removed. Is it possible that the heat produced by the friction may have assisted the process? The violet colour of the lips of the furrow may result from pressure after death, but this must have been exerted within a very few hours, and even then it will rarely be observed upon the lower border. Esquirol was the first to take notice of a bright silvery line in the subcutaneous cellular membrane of the furrow. It is a purely mechanical change, resulting from the condensation of this tissue, and will present a more or less glistening appearance according to the quantity of fluid contained in the part.

The most unequivocal signs of suspension during life are, extravasation in the cellular membrane and in the muscles, lacerations of these parts, fractures of the os hyoides and laryngeal cartilages, and rupture of the vertebral ligaments; but it is necessary that these several solutions of continuity should be accompanied by true ecchymosis. M. Amussat first noticed the curious fact, that the inner and middle coats of the common carotids may be divided by a cord applied during life. Devergie has met with one case confirmatory of this observation; and, by several experiments, he has proved that the injury cannot be imitated in the dead body. If to the above signs be added the traces of emissio seminis, and of urinary discharge, we have enumerated all the indications that can be relied upon with certainty in proof of strangulation during life; but they may all be absent, notwithstanding the individual died from this cause. It is highly improbable, however, that they should be wanting when the individual has been strangled by violence. We are somewhat surprised at the following observations of the author.

"All the cases in which the signs are least decided belong to suicide, and in these the moral proofs are often so convincing that an autopsy is a work of supererogation. Moreover, magistrates never give orders for it. During the seven years that I have been attached to the Morgue, where there are annually deposited twenty bodies killed by hanging, there has not been, to my knowledge, a single case of medico-legal dissection. The reason is simple: a murderer would rarely be disposed to sacrifice his victim by a method so difficult of achievement."

It seems to us that if the signs are so likely to be wanting in cases of suicide, this circumstance might be taken advantage of by the murderer who has despatched his victim by other means, and wishes to make it appear that the latter had himself put an end to his existence. It is scarcely a far-fetched conception to imagine that a person might poison another by a strong dose of prussic acid, administered perhaps during sleep, and suspend the body immediately afterwards. In such a case, if there were any moral presumptions of a suicidal tendency on the part of the deceased, it would be difficult to come at the truth: at all events, an examination after death would be any thing but "*un surcroit d'investigation.*"

We now take our leave of M. Devergie; but with the intention of resuming the survey of his work at a future opportunity; probably when it is quite completed. He has yet to grapple with two great subjects, Insanity and Toxicology, besides others of minor interest. Should he prove as successful in the management of these topics as of those which have already engaged his pen, we shall have but little hesitation in pronouncing his treatise to be the best guide to the student of forensic medicine that the French language, rich as it is in works of this description, can afford. Not that in execution it is superior or even equal to the writings of Mahon, Fodéré, and Orfila, but that the information which it contains is derived from the most recent as well as the best established discoveries in medical science.

Our attention must now be directed to Mr. TAYLOR's Elements. The credit of our national literature required the appearance of a publication comprising the later improvements, which have been very numerous and important since the issue of the respective works of Drs. Male, Smith, and Paris. To Dr. Beck's admirable treatise, a new edition of which has recently been published, with an abundance of fresh matter, worthy of the stock to which it has been added, our literature can lay no claim; though Englishmen must feel proud that such a work should have been written in their language. Mr. Taylor's treatise, as we have already remarked, has the merit of presenting a fair view of the present state of the science. We cannot say that the author has brought forward many researches peculiar to himself, but he has compiled well; by which we imply, that his matter is sound, ample, well arranged, and, above all, adapted to practical purposes.

We think that here and there he has somewhat neglected to enrich his illustrations with gleanings from foreign fields, but the occasions are rare in which anything is omitted that would tend materially to the guidance of the medical jurist in difficult questions.

Before proceeding to select one or two subjects for comment, we must beg leave to express our regret that Mr. Taylor should have assisted, by the title of his book, in keeping up the use of the term Medical Jurisprudence. If there were no other designation for the science, it might perhaps admit of question whether a new one should be created, as no kind of plurality is more objectionable than that of names: but, when a more correct and appropriate term is in common use, we are surprised that a teacher of the science should not adopt it. If Forensic Medicine is a fitting designation of the science to which it is applied, (which we never found any one inclined to dispute,) it is obvious that medical jurisprudence cannot also be proper. The latter signifies the knowledge of law as applicable to medicine, which, as an art or application of science, may be affected by law, as in medical police, and likewise as a *profession*, but not as a science, unless the laws of nature are amenable to human regulations. Forensic medicine signifies that department of medicine, considered either as a science or an art, which is applicable to law, whether legislative or administrative. If we have correctly defined the respective meanings of these terms, there cannot be a moment's question as to which ought to be employed by medico-legal writers.

Although conciseness is one of the characteristics of the work, viewed as a whole, the first chapter, which treats of Asphyxia, is certainly unnecessarily prolix. In a purely physiological work, some extent of speculation is allowable, even upon a subject which is still in need of demonstrative evidence; but, in a treatise from which we hope to derive helps to practice, hypothetical excursions are impertinent. After a few preliminary observations upon the external causes and the phænomena of asphyxia, the author enters upon two principal inquiries: "1st. The nature of the change which the blood circulated in asphyxia is stated to undergo; 2d, the probable influence of this fluid upon the organs of the body."

He admits that the arrest of the circulation takes place in the capillaries of the lungs; a point which our readers are doubtless aware Dr. Williams and Dr. Kay have established by experiments. He takes great pains to prove the incorrectness of Dr. Kay's views relative to the mode in which the pulmonary obstruction is effected. Dr. Kay assumes that the capillaries of the pulmonary veins are endowed with a peculiar organic sensibility, which indisposes them to admit unarterialized blood. The circulation of dark blood after the commencement of asphyxia he considers not irreconcileable with this view, because there remains in the lungs a certain quantity of oxygen after the stoppage of the respiratory action, which serves for a short time to arterialize the blood, though imperfectly; and he therefore thinks that the dark blood is not actually venous, but only imperfectly oxygenized. After a certain period, generally about three minutes, blood ceases to flow from the pulmonary veins, and the action of the heart is arrested, not because this organ has lost its contractile power, but because the blood is not delivered to the left side in sufficient quantity to maintain its contractions. Mr. Taylor will not allow the venous radicles in the lungs to possess any such sensibility as Dr. Kay supposes. This he is quite at liberty to doubt, since it must necessarily be a matter of pure conjecture; but, when he expresses an opinion that there is no proof of any difficulty to the transmission of venous blood, *quà* venous, through the pulmonary capillaries, (setting aside the *cause* of the difficulty,) we think him decidedly in error. He alludes triumphantly to the results of some other experiments of Dr. Kay himself, in which venous blood, when injected into an artery, was found to maintain the contractility of muscles, and urges that, if it circulated through the capillaries of these parts, there is no reason for presuming the existence of any difficulty in the lungs. We are surprised that he should not have considered the probability that the pulmonary capillaries would be particularly guarded, in some way or other, against the entrance of venous blood; since, if this could take place with facility, the tissues would often, from a variety of causes, incur the risk of receiving but an indifferent supply of oxygenized blood. He imagines that the dark blood, which is circulated after the stoppage of the respiration, has not been at all acted upon by oxygen; for he is unwilling to allow that what gas may remain in the lungs produces the effect ascribed to it by Dr. Kay; for which denial he gives no other reason than that it has not been proved experimentally that the air found in the lungs of asphyxiated animals is deprived of all its oxygen. The fact is, the author has an hypothesis of his own, which he thinks a much better explanation of the matter. The blood, he says, appears to be gradually vitiated, not because it undergoes alteration as long as oxygen continues in the lungs, but because the venous blood is mixed with the red blood circulating in the arteries at the commencement of asphyxia. Now, to our apprehension, this mixture is not only not proved, but is even inconceivable, except in the cavities of the heart itself. In the vessels where Mr. Taylor imagines the process to occur, we consider it physically impossible; for, supposing red blood were in the aorta at the time that black blood issues from the left ventricle, it is obvious that, in the continuous stream of the circulation, the red blood will not wait to be tinctured by the black, but that the one will follow the other. But let us concede for a moment to the author that the obstruction in the lungs is not caused by the quality of the blood, in order that we may learn what he considers to be the impeding agents. These, after a few remarks upon the physiology of the pulmonary circulation during respiration, he concludes to be the collapsed state of the lungs and the enfeebled action of the right ventricle, and possibly a change in the innervation of the capillaries of the lungs by sympathy with the brain. We shall not stop to combat this revived error of Haller's respecting the supposed mechanical impediment to the pulmonary circulation, as we think that the arguments and experiments of Goodwin have completely set that question at rest. "Besides," as Dr. Alison remarks, "we know that the same stagnation in the lungs takes place in the case of an animal confined in a gas which does not contain free oxygen, as in the case of drowning or strangulation, although, in the former case, any impediment to the mechanical acts of respiration that can occur must be the consequence, not the cause, of the fatal changes within the chest."

The heart may be enfeebled, for aught we can tell to the contrary; but if its defective contraction were the cause of the accumulation in the lungs, how are we to explain the reviving influence of readmitted oxygen? If the heart has ceased to beat because its power of contracting is lost, and the pulmonary arteries are gorged because the right ventricle has failed to propel the blood through them, the contact of air with the blood would be incapable of restoring the circulation, because it could have no effect upon the heart. We really think that physiologists have given themselves some unnecessary trouble upon the subject. If they had simply observed that stagnation, more or less complete, in the pulmonary capillaries, takes place upon the interception, and is removed upon the readmission of air, and upon the consequent alteration of the blood, it would have been an all but necessary inference that the want of this change had caused the stagnation. That this is the case we have long believed, but our conviction has been materially strengthened by some extremely ingenious observations communicated by Dr. Alison to the British Association, and since embodied in his article on *Asphyxia* in the Cyclopædia of Anatomy; a dissertation which, we imagine, Mr. Taylor cannot have met with, or he would scarcely have omitted to notice it, even if it had not tended considerably to alter his views upon the subject of asphyxia.

We shall not follow the author in his prosecution of the inquiry whether venous blood extinguishes the contractility of muscle and the function of the brain. It can require no proof that the unarterialized blood which he believes to circulate, or the imperfectly arterialized fluid of Dr. Kay, must be unfitted for the function of any part; or why should respiration be necessary? But before the cerebral derangement can materially influence the circulation, this function has been stopped by the cause above mentioned. The cessation of the animal functions is perhaps due in some measure to the vitiated blood; but it seems quite as probable that the impeded circulation in the lungs, which must operate by the obstacle presented to the return of the blood from the brain, may have something to do with the result.

In the second chapter we have a sufficient account of the Signs of Real and Apparent Death, a subject upon which many medico-legal writers have been needlessly liberal of their instructions. We must observe, however, that Mr. Taylor has omitted, in his remarks upon Rigidity, to take notice of that kind of stiffness which sometimes remains after death by diseases of the nervous system and by asphyxia, and which it is highly important to distinguish from true cadaveric rigidity. It is well known that the latter appears very late in cases of asphyxia; on which account Orfila reasonably infers that, if the limbs are found stiff while the animal heat remains but little impaired, in a person who has died asphyxiated, the death must have been very recent; the rigidity being spasmodic, not cadaveric. (*Lec. de Méd. Lég.*, II., 195.) The importance of bearing this distinction in mind will be obvious to any one who has read M. Marc's medico-legal discussion of the case of Jean Courbon (*Ann. d'Hyg.* vii., 604;) and Dr. Fletcher's very interesting account of the trial of Robert Reid for murder of his wife. Mr. Taylor refers his readers for the original of the facies Hippocratica, to the fifth section of the second Book de Morbis. This reference is also given by nearly all the French writers, who appear to have borrowed it from one another. Indications of death from the countenance are scattered here and there through the treatise, but in no part of it is there such a summary as they have given, nor any thing like it. The nearest approach to the description will be found in the first part of the "Prænotiones," but even there the signs are not grouped into such a portrait as Mr. Taylor and his predecessors have depicted.

Death by Drowning is very ably treated of in the third chapter.

The author has selected several interesting illustrations from trials which have occurred in our own courts within the last few years, of some of the more embarrassing questions connected with this subject. The following account of some experiments performed by himself to determine under what circumstances water is most likely to be found in the stomach of a drowned person are worthy of attention.

"We allow, then, that water may enter into the stomach of a drowning animal, but in variable quantity; and we must, at the same time, admit that there are cases of drowning in which water is not discovered in the organ. In dissecting cats which had been drowned, I have repeatedly remarked the absence of water from the stomach: in these cases, the animals had been invariably kept under water from the first moment of their submersion, and thus in a condition but little favorable to the exercise of deglutition. Water does not readily penetrate into the stomach of a subject which has been thrown in after death, the parietes of the œsophagus applying themselves too closely to each other to allow of the passage of the fluid. If putrefaction has advanced to any extent, it is possible that water may enter: but the practitioner will easily judge from the state of the body how far this process may have been concerned in the admission of the fluid into the stomach and alimentary canal. It is, however, necessary for the Medical jurist to know that there are cases in which water may be found in the stomachs of persons apparently drowned and yet it will afford no evidence of death having been caused by drowning. Thus, if a body be sunk to a very great depth in water, this fluid will find its way into the stomach and alimentary canal by virtue of its columnar pressure. In order to ascertain how far columnar pressure would influence the quantity of water contained within the stomach of a drowned animal, I performed the following experiments: Three cats, of nearly equal size, were taken: No. 1 was rapidly lowered to the depth of fifty-five feet in the Thames; No. 2 was lowered to the depth of two feet below the surface of the water, being forcibly maintained in that position; whilst No. 3 was drowned on the surface, but was allowed to sink and rise to respire frequently before death. The three cats were removed from the water after the lapse of a quarter of an hour, and, on examination, it was discovered that the stomach of No. 1 was completely distended with water; that little or no water was contained in that of No. 2; but that the stomach of No. 3 was filled, although not to so great an extent as that of No. 1. By comparing the results obtained in the first two cases, we must be allowed to infer that the depth to which an animal sinks in drowning may affect materially the quantity of water found within its stomach. The influence of this columnar pressure may be conceived to act on the body, whether it be submerged living or dead; and, therefore, it will be proper that the witness should take this into consideration before he positively decides from the discovery of water in this organ that death has taken place by drowning." (P. 120-121.)

We are surprised that, when speaking of putrefaction in water, Mr. Taylor should have taken no notice of the labours of Devergie. It is very possible that this part of the volume was written before the publication of the work reviewed in the present article; but the researches alluded to have long been presented to the profession, and been the subject of controversy in the French journals.

The chapter on Hanging, Strangulation, and Suffocation, may upon the whole be safely trusted to as a digest of the best information upon these subjects. We think, however, that the researches of Remer, and those of Fleichmann, ought to have been mentioned and commented upon. When treating of suicidal strangling, the author might have collected more instances than he has done in proof of the possibility of the occurrence, which has often been disputed.

The fifth chapter is devoted to Lightning, Cold, Starvation, and Fire, and contains some very valuable matter. But, by far the most important part of the volume is that which treats of *Wounds*, and which occupies the last four chapters. Mr. T. has shewn great judgment in assigning so large a space to this subject. "Trials for murder and manslaughter by wounding," as he remarks in his preface, "are very frequent in our courts of law; and I flatter myself that the copious selection of modern cases which are dispersed through the concluding chapters of the volume will point out to the practitioner those questions which he is most commonly required to answer, and for the elucidation of which medical evidence is indispensable." We cannot praise too highly the great pains and earnestness with which the author directs the attention of practitioners to the great variety of questions that are involved in some of the most common casualties. In every part of the discussion he manifests a thorough practical acquaintance with his subject, and we regret that our space obliges us to speak of his labours in general terms only. Whether in distinguishing injuries of the living body from cadaveric lesions, or in deciding upon the medico-legal character of wounds by the consideration of circumstances that may have occurred prior or subsequently to the wound, or of the nature of the wound itself, or of the part in which it occurs, he is equally correct in his statements, happy in his illustrations, and prudent in his admonitions. We must find room for an account of some experiments intended to elucidate a very important question.

"The following experiments to determine the characters of an incised wound inflicted *after death*, were undertaken by a friend and myself in the summer of 1832. Opportunities but rarely present themselves where such inquiries can be pursued upon the human body so soon after dissolution as the nature of the investigation requires. In consequence of this obstacle, we selected some limbs immediately after amputation, and the results obtained by experimenting on these limbs will, I think, suffice to give us an idea of those which would follow similar experiments performed on the recently dead subject.

"In the first, an incised wound, about three inches in length, was made in the upper part of the calf of the leg *two minutes* after its separation from the body, by which the gastrocnemii muscles and the fascia covering the deep-seated layer of the leg were divided. At the moment that the wound was made, the skin retracted considerably, causing a protrusion of the adipose substance beneath, the quantity of blood which escaped was small, the cellular membrane by its sudden protrusion forwards seeming mechanically to prevent its exit. The wound was examined after the lapse of twenty-four hours:—the edges were red, bloody, and everted, the skin was not in the least degree tumefied, but merely somewhat flaccid. On separating the edges, a small quantity of fluid blood escaped, but no coagula were seen adhering to the muscles. At the bottom of the wound, however, and in close contact with the fascia, we found a small quantity of coagulated blood; but the coagula were so loose as readily to break down under the finger.

"In the second experiment, an incision of similar extent was made on the outer side of the leg, penetrating through the peronei and into the flexor longus pollicis of the deep-seated layer of muscles, *ten minutes* after the separation of the member from the body. In this case, the skin appeared to have lost its elasticity; for the edges of the wound became but very slightly everted; scarcely any blood escaped from it. On examining the leg twenty-four hours afterwards, the edges of the incision were pale and perfectly collapsed, presenting none of the characters of a wound inflicted during life. Still, at the bottom of the wound, and enclosed by the divided muscular fibres, we met with some coagula of blood; but these were certainly fewer than in the former experiment. A portion of liquid blood had evidently escaped, owing to the leg having been moved.

"Other experiments were performed at a still later period after the removal of the limbs; and it was found that, in proportion to the length of time suffered to elapse before the production of the wound, so were the appearances less distinctly marked; that is to say, the less likely were they to be confounded with similar injuries inflicted upon the living body. When the incised wound was not made until *two or three hours* after the removal of the limb, although a small quantity of liquid blood was effused, no coagula were found. The edges of an incised wound made twenty-four hours after death will be found yielding, inelastic, in close approximation, and, as far as our observations extend, free from any coagula of blood." (P. 275.)

We do not apologize to Mr. Taylor for having devoted a smaller space to his treatise than to the work of M. Devergie, because he will at once perceive that the latter is far less likely than his own to fall into the hands of English readers. We conclude, however, with a remark somewhat similar to that which terminated our examination of Devergie's treatise; namely, that, if some of the remaining subjects, such as Infanticide, Insanity, and Poisoning, should be treated with the same care and ability in the second volume as the author has already manifested, we shall have no difficulty in pronouncing Mr. Taylor's "Elements" to be, with one exception in favour of Beck's treatise, the most useful compendium of medico-legal science to which the English practitioner or student can have recourse.

